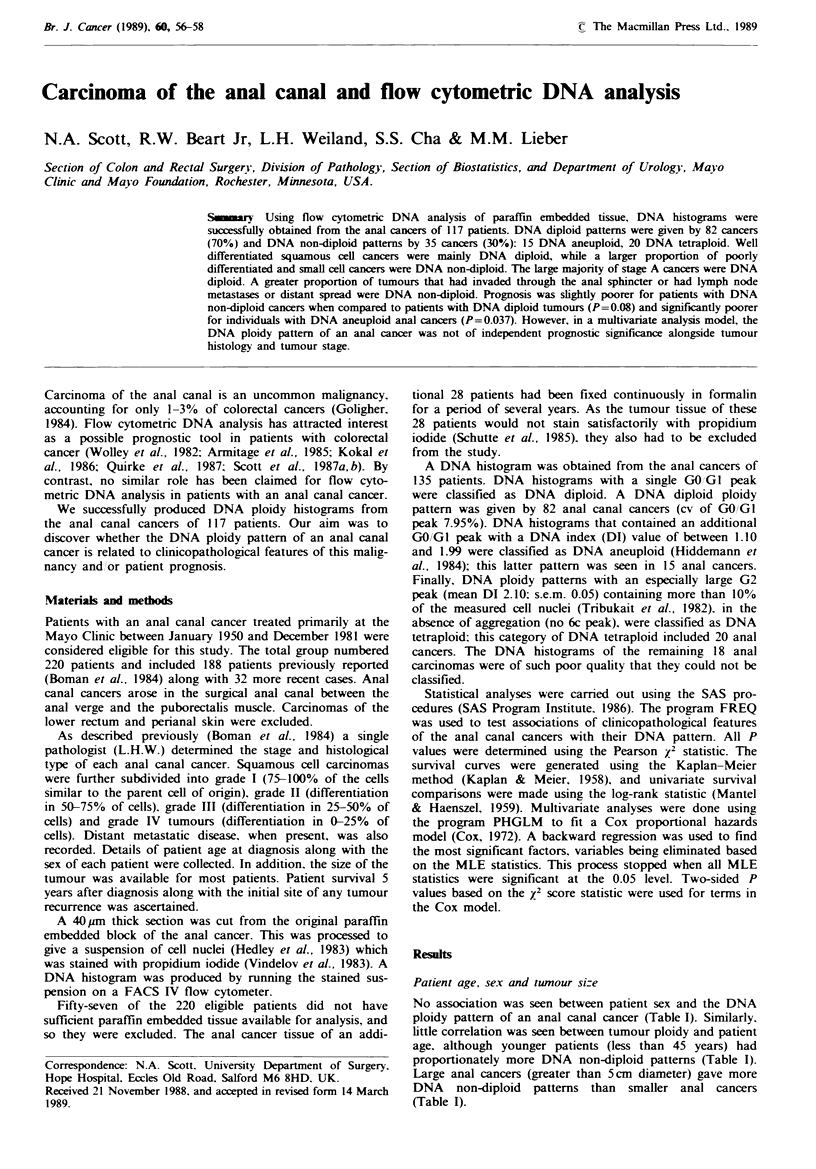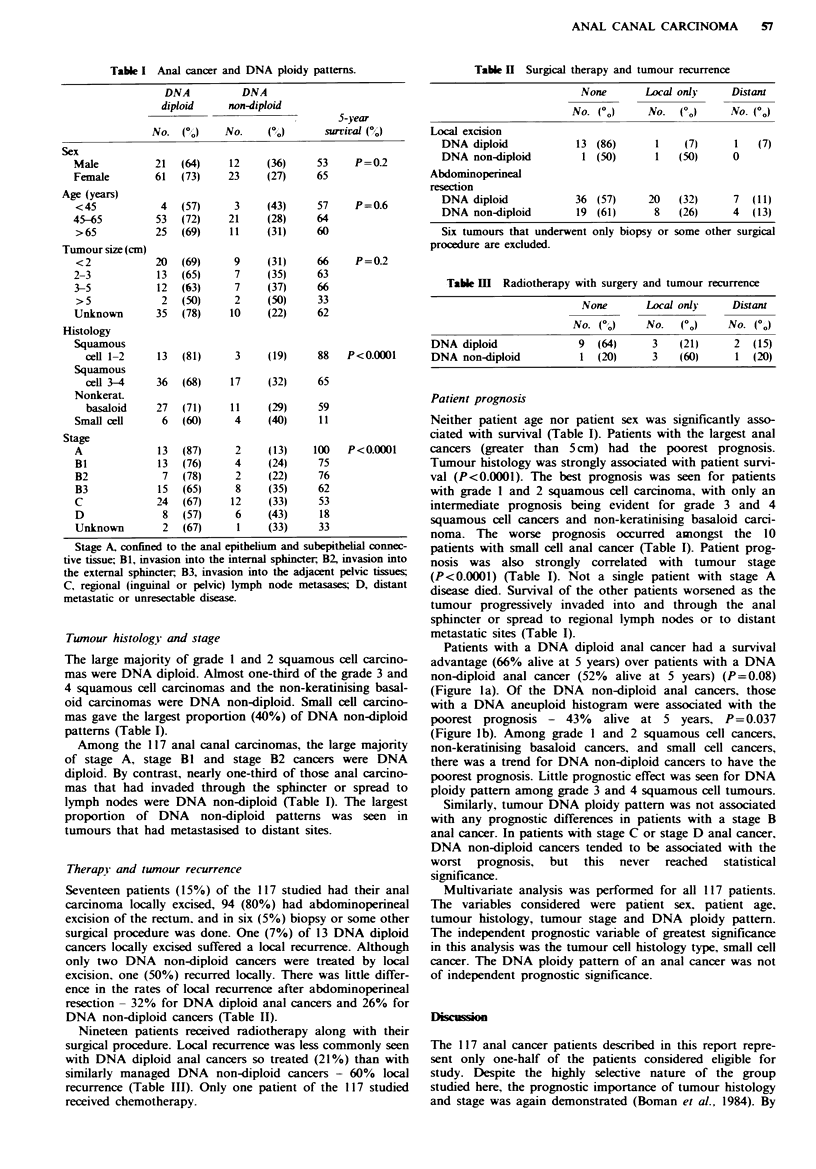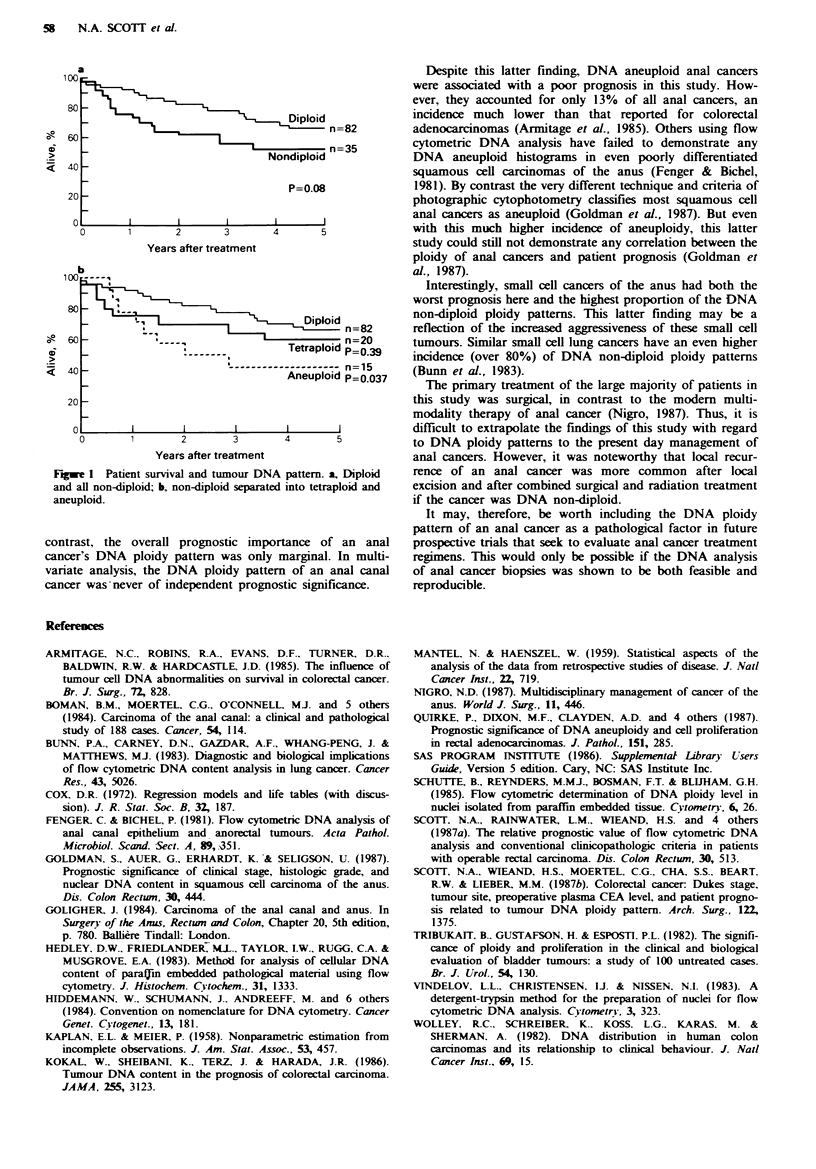# Carcinoma of the anal canal and flow cytometric DNA analysis.

**DOI:** 10.1038/bjc.1989.219

**Published:** 1989-07

**Authors:** N. A. Scott, R. W. Beart, L. H. Weiland, S. S. Cha, M. M. Lieber

**Affiliations:** Division of Pathology, Mayo Clinic, Rochester, Minnesota.

## Abstract

Using flow cytometric DNA analysis of paraffin embedded tissue, DNA histograms were successfully obtained from the anal cancers of 117 patients. DNA diploid patterns were given by 82 cancers (70%) and DNA non-diploid patterns by 35 cancers (30%): 15 DNA aneuploid, 20 DNA tetraploid. Well differentiated squamous cell cancers were mainly DNA diploid, while a larger proportion of poorly differentiated and small cell cancers were DNA non-diploid. The large majority of stage A cancers were DNA diploid. A greater proportion of tumours that had invaded through the anal sphincter or had lymph node metastases or distant spread were DNA non-diploid. Prognosis was slightly poorer for patients with DNA non-diploid cancers when compared to patients with DNA diploid tumours (P = 0.08) and significantly poorer for individuals with DNA aneuploid anal cancers (P = 0.037). However, in a multivariate analysis model, the DNA ploidy pattern of an anal cancer was not of independent prognostic significance alongside tumour histology and tumour stage.


Br. J. Cancer (1989), 60, 56-58 © The Macmillan Press Ltd., 1989

### Carcinoma of the anal canal and flow cytometric DNA analysis

N.A. Scott, R.W. Beart Jr, L.H. Weiland, S.S. Cha & M.M. Lieber

*Section of Colon and Rectal Surgery, Division of Pathology, Section of Biostatistics, and Department of Urology, Mayo Clinic and Mayo Foundation, Rochester, Minnesota, USA.*

**Summary** Using flow cytometric DNA analysis of paraffin embedded tissue, DNA histograms were successfully obtained from the anal cancers of 117 patients. DNA diploid patterns were given by 82 cancers (70%) and DNA non-diploid patterns by 35 cancers (30%): 15 DNA aneuploid, 20 DNA tetraploid. Well differentiated squamous cell cancers were mainly DNA diploid, while a larger proportion of poorly differentiated and small cell cancers were DNA non-diploid. The large majority of stage A cancers were DNA diploid. A greater proportion of tumours that had invaded through the anal sphincter or had lymph node metastases or distant spread were DNA non-diploid. Prognosis was slightly poorer for patients with DNA non-diploid cancers when compared to patients with DNA diploid tumours ($P=0.08$) and significantly poorer for individuals with DNA aneuploid anal cancers ($P=0.037$). However, in a multivariate analysis model, the DNA ploidy pattern of an anal cancer was not of independent prognostic significance alongside tumour histology and tumour stage.

Carcinoma of the anal canal is an uncommon malignancy, accounting for only 1–3% of colorectal cancers (Goligher, 1984). Flow cytometric DNA analysis has attracted interest as a possible prognostic tool in patients with colorectal cancer (Wolley *et al.*, 1982; Armitage *et al.*, 1985; Kokal *et al.*, 1986; Quirke *et al.*, 1987; Scott *et al.*, 1987*a,b*). By contrast, no similar role has been claimed for flow cytometric DNA analysis in patients with an anal canal cancer.

We successfully produced DNA ploidy histograms from the anal canal cancers of 117 patients. Our aim was to discover whether the DNA ploidy pattern of an anal canal cancer is related to clinicopathological features of this malignancy and/or patient prognosis.

### Materials and methods

Patients with an anal canal cancer treated primarily at the Mayo Clinic between January 1950 and December 1981 were considered eligible for this study. The total group numbered 220 patients and included 188 patients previously reported (Boman *et al.*, 1984) along with 32 more recent cases. Anal canal cancers arose in the surgical anal canal between the anal verge and the puborectalis muscle. Carcinomas of the lower rectum and perianal skin were excluded.

As described previously (Boman *et al.*, 1984) a single pathologist (L.H.W.) determined the stage and histological type of each anal canal cancer. Squamous cell carcinomas were further subdivided into grade I (75–100% of the cells similar to the parent cell of origin), grade II (differentiation in 50–75% of cells), grade III (differentiation in 25–50% of cells) and grade IV tumours (differentiation in 0–25% of cells). Distant metastatic disease, when present, was also recorded. Details of patient age at diagnosis along with the sex of each patient were collected. In addition, the size of the tumour was available for most patients. Patient survival 5 years after diagnosis along with the initial site of any tumour recurrence was ascertained.

A 40 $\mu$m thick section was cut from the original paraffin embedded block of the anal cancer. This was processed to give a suspension of cell nuclei (Hedley *et al.*, 1983) which was stained with propidium iodide (Vindelov *et al.*, 1983). A DNA histogram was produced by running the stained suspension on a FACS IV flow cytometer.

Fifty-seven of the 220 eligible patients did not have sufficient paraffin embedded tissue available for analysis, and so they were excluded. The anal cancer tissue of an additional 28 patients had been fixed continuously in formalin for a period of several years. As the tumour tissue of these 28 patients would not stain satisfactorily with propidium iodide (Schutte *et al.*, 1985), they also had to be excluded from the study.

A DNA histogram was obtained from the anal cancers of 135 patients. DNA histograms with a single G0/G1 peak were classified as DNA diploid. A DNA diploid ploidy pattern was given by 82 anal canal cancers (cv of G0/G1 peak 7.95%). DNA histograms that contained an additional G0/G1 peak with a DNA index (DI) value of between 1.10 and 1.99 were classified as DNA aneuploid (Hiddemann *et al.*, 1984); this latter pattern was seen in 15 anal cancers. Finally, DNA ploidy patterns with an especially large G2 peak (mean DI 2.10; s.e.m. 0.05) containing more than 10% of the measured cell nuclei (Tribukait *et al.*, 1982), in the absence of aggregation (no 6c peak), were classified as DNA tetraploid; this category of DNA tetraploid included 20 anal cancers. The DNA histograms of the remaining 18 anal carcinomas were of such poor quality that they could not be classified.

Statistical analyses were carried out using the SAS procedures (SAS Program Institute, 1986). The program FREQ was used to test associations of clinicopathological features of the anal canal cancers with their DNA pattern. All $P$ values were determined using the Pearson $\chi^2$ statistic. The survival curves were generated using the Kaplan–Meier method (Kaplan & Meier, 1958), and univariate survival comparisons were made using the log-rank statistic (Mantel & Haenszel, 1959). Multivariate analyses were done using the program PHGLM to fit a Cox proportional hazards model (Cox, 1972). A backward regression was used to find the most significant factors, variables being eliminated based on the MLE statistics. This process stopped when all MLE statistics were significant at the 0.05 level. Two-sided $P$ values based on the $\chi^2$ score statistic were used for terms in the Cox model.

### Results

### *Patient age, sex and tumour size*

No association was seen between patient sex and the DNA ploidy pattern of an anal canal cancer (Table I). Similarly, little correlation was seen between tumour ploidy and patient age, although younger patients (less than 45 years) had proportionately more DNA non-diploid patterns (Table I). Large anal cancers (greater than 5 cm diameter) gave more DNA non-diploid patterns than smaller anal cancers (Table I).

Correspondence: N.A. Scott, University Department of Surgery, Hope Hospital, Eccles Old Road, Salford M6 8HD, UK.
Received 21 November 1988, and accepted in revised form 14 March 1989.

**Table I** Anal cancer and DNA ploidy patterns.

| | DNA diploid | | DNA non-diploid | | 5-year survival (%) | |
|---|---|---|---|---|---|---|
| | No. | (%) | No. | (%) | | |
| **Sex** | | | | | | |
| Male | 21 | (64) | 12 | (36) | 53 | $P=0.2$ |
| Female | 61 | (73) | 23 | (27) | 65 | |
| **Age (years)** | | | | | | |
| <45 | 4 | (57) | 3 | (43) | 57 | $P=0.6$ |
| 45–65 | 53 | (72) | 21 | (28) | 64 | |
| >65 | 25 | (69) | 11 | (31) | 60 | |
| **Tumour size (cm)** | | | | | | |
| <2 | 20 | (69) | 9 | (31) | 66 | $P=0.2$ |
| 2–3 | 13 | (65) | 7 | (35) | 63 | |
| 3–5 | 12 | (63) | 7 | (37) | 66 | |
| >5 | 2 | (50) | 2 | (50) | 33 | |
| Unknown | 35 | (78) | 10 | (22) | 62 | |
| **Histology** | | | | | | |
| Squamous cell 1–2 | 13 | (81) | 3 | (19) | 88 | $P<0.0001$ |
| Squamous cell 3–4 | 36 | (68) | 17 | (32) | 65 | |
| Nonkerat. basaloid | 27 | (71) | 11 | (29) | 59 | |
| Small cell | 6 | (60) | 4 | (40) | 11 | |
| **Stage** | | | | | | |
| A | 13 | (87) | 2 | (13) | 100 | $P<0.0001$ |
| B1 | 13 | (76) | 4 | (24) | 75 | |
| B2 | 7 | (78) | 2 | (22) | 76 | |
| B3 | 15 | (65) | 8 | (35) | 62 | |
| C | 24 | (67) | 12 | (33) | 53 | |
| D | 8 | (57) | 6 | (43) | 18 | |
| Unknown | 2 | (67) | 1 | (33) | 33 | |

Stage A, confined to the anal epithelium and subepithelial connective tissue; B1, invasion into the internal sphincter; B2, invasion into the external sphincter; B3, invasion into the adjacent pelvic tissues; C, regional (inguinal or pelvic) lymph node metasases; D, distant metastatic or unresectable disease.

### *Tumour histology and stage*

The large majority of grade 1 and 2 squamous cell carcinomas were DNA diploid. Almost one-third of the grade 3 and 4 squamous cell carcinomas and the non-keratinising basaloid carcinomas were DNA non-diploid. Small cell carcinomas gave the largest proportion (40%) of DNA non-diploid patterns (Table I).

Among the 117 anal canal carcinomas, the large majority of stage A, stage B1 and stage B2 cancers were DNA diploid. By contrast, nearly one-third of those anal carcinomas that had invaded through the sphincter or spread to lymph nodes were DNA non-diploid (Table I). The largest proportion of DNA non-diploid patterns was seen in tumours that had metastasised to distant sites.

### *Therapy and tumour recurrence*

Seventeen patients (15%) of the 117 studied had their anal carcinoma locally excised, 94 (80%) had abdominoperineal excision of the rectum, and in six (5%) biopsy or some other surgical procedure was done. One (7%) of 13 DNA diploid cancers locally excised suffered a local recurrence. Although only two DNA non-diploid cancers were treated by local excision, one (50%) recurred locally. There was little difference in the rates of local recurrence after abdominoperineal resection – 32% for DNA diploid anal cancers and 26% for DNA non-diploid cancers (Table II).

Nineteen patients received radiotherapy along with their surgical procedure. Local recurrence was less commonly seen with DNA diploid anal cancers so treated (21%) than with similarly managed DNA non-diploid cancers – 60% local recurrence (Table III). Only one patient of the 117 studied received chemotherapy.

**Table II** Surgical therapy and tumour recurrence

| | None | | Local only | | Distant | |
|---|---|---|---|---|---|---|
| | No. | (%) | No. | (%) | No. | (%) |
| Local excision | | | | | | |
| DNA diploid | 13 | (86) | 1 | (7) | 1 | (7) |
| DNA non-diploid | 1 | (50) | 1 | (50) | 0 | |
| Abdominoperineal resection | | | | | | |
| DNA diploid | 36 | (57) | 20 | (32) | 7 | (11) |
| DNA non-diploid | 19 | (61) | 8 | (26) | 4 | (13) |

Six tumours that underwent only biopsy or some other surgical procedure are excluded.

**Table III** Radiotherapy with surgery and tumour recurrence

| | None | | Local only | | Distant | |
|---|---|---|---|---|---|---|
| | No. | (%) | No. | (%) | No. | (%) |
| DNA diploid | 9 | (64) | 3 | (21) | 2 | (15) |
| DNA non-diploid | 1 | (20) | 3 | (60) | 1 | (20) |

### *Patient prognosis*

Neither patient age nor patient sex was significantly associated with survival (Table I). Patients with the largest anal cancers (greater than 5 cm) had the poorest prognosis. Tumour histology was strongly associated with patient survival ($P<0.0001$). The best prognosis was seen for patients with grade 1 and 2 squamous cell carcinoma, with only an intermediate prognosis being evident for grade 3 and 4 squamous cell cancers and non-keratinising basaloid carcinoma. The worse prognosis occurred amongst the 10 patients with small cell anal cancer (Table I). Patient prognosis was also strongly correlated with tumour stage ($P<0.0001$) (Table I). Not a single patient with stage A disease died. Survival of the other patients worsened as the tumour progressively invaded into and through the anal sphincter or spread to regional lymph nodes or to distant metastatic sites (Table I).

Patients with a DNA diploid anal cancer had a survival advantage (66% alive at 5 years) over patients with a DNA non-diploid anal cancer (52% alive at 5 years) ($P=0.08$) (Figure 1a). Of the DNA non-diploid anal cancers, those with a DNA aneuploid histogram were associated with the poorest prognosis – 43% alive at 5 years, $P=0.037$ (Figure 1b). Among grade 1 and 2 squamous cell cancers, non-keratinising basaloid cancers, and small cell cancers, there was a trend for DNA non-diploid cancers to have the poorest prognosis. Little prognostic effect was seen for DNA ploidy pattern among grade 3 and 4 squamous cell tumours.

Similarly, tumour DNA ploidy pattern was not associated with any prognostic differences in patients with a stage B anal cancer. In patients with stage C or stage D anal cancer, DNA non-diploid cancers tended to be associated with the worst prognosis, but this never reached statistical significance.

Multivariate analysis was performed for all 117 patients. The variables considered were patient sex, patient age, tumour histology, tumour stage and DNA ploidy pattern. The independent prognostic variable of greatest significance in this analysis was the tumour cell histology type, small cell cancer. The DNA ploidy pattern of an anal cancer was not of independent prognostic significance.

### Discussion

The 117 anal cancer patients described in this report represent only one-half of the patients considered eligible for study. Despite the highly selective nature of the group studied here, the prognostic importance of tumour histology and stage was again demonstrated (Boman *et al.*, 1984). By

**Figure 1** Patient survival and tumour DNA pattern. **a**, Diploid and all non-diploid; **b**, non-diploid separated into tetraploid and aneuploid.

contrast, the overall prognostic importance of an anal cancer's DNA ploidy pattern was only marginal. In multivariate analysis, the DNA ploidy pattern of an anal canal cancer was never of independent prognostic significance.

Despite this latter finding, DNA aneuploid anal cancers were associated with a poor prognosis in this study. However, they accounted for only 13% of all anal cancers, an incidence much lower than that reported for colorectal adenocarcinomas (Armitage et al., 1985). Others using flow cytometric DNA analysis have failed to demonstrate any DNA aneuploid histograms in even poorly differentiated squamous cell carcinomas of the anus (Fenger & Bichel, 1981). By contrast the very different technique and criteria of photographic cytophotometry classifies most squamous cell anal cancers as aneuploid (Goldman et al., 1987). But even with this much higher incidence of aneuploidy, this latter study could still not demonstrate any correlation between the ploidy of anal cancers and patient prognosis (Goldman et al., 1987).

Interestingly, small cell cancers of the anus had both the worst prognosis here and the highest proportion of the DNA non-diploid ploidy patterns. This latter finding may be a reflection of the increased aggressiveness of these small cell tumours. Similar small cell lung cancers have an even higher incidence (over 80%) of DNA non-diploid ploidy patterns (Bunn et al., 1983).

The primary treatment of the large majority of patients in this study was surgical, in contrast to the modern multimodality therapy of anal cancer (Nigro, 1987). Thus, it is difficult to extrapolate the findings of this study with regard to DNA ploidy patterns to the present day management of anal cancers. However, it was noteworthy that local recurrence of an anal cancer was more common after local excision and after combined surgical and radiation treatment if the cancer was DNA non-diploid.

It may, therefore, be worth including the DNA ploidy pattern of an anal cancer as a pathological factor in future prospective trials that seek to evaluate anal cancer treatment regimens. This would only be possible if the DNA analysis of anal cancer biopsies was shown to be both feasible and reproducible.

### References

ARMITAGE, N.C., ROBINS, R.A., EVANS, D.F., TURNER, D.R., BALDWIN, R.W. & HARDCASTLE, J.D. (1985). The influence of tumour cell DNA abnormalities on survival in colorectal cancer. *Br. J. Surg.*, **72**, 828.

BOMAN, B.M., MOERTEL, C.G., O'CONNELL, M.J. and 5 others (1984). Carcinoma of the anal canal: a clinical and pathological study of 188 cases. *Cancer*, **54**, 114.

BUNN, P.A., CARNEY, D.N., GAZDAR, A.F., WHANG-PENG, J. & MATTHEWS, M.J. (1983). Diagnostic and biological implications of flow cytometric DNA content analysis in lung cancer. *Cancer Res.*, **43**, 5026.

COX, D.R. (1972). Regression models and life tables (with discussion). *J. R. Stat. Soc. B*, **32**, 187.

FENGER, C. & BICHEL, P. (1981). Flow cytometric DNA analysis of anal canal epithelium and anorectal tumours. *Acta Pathol. Microbiol. Scand. Sect. A*, **89**, 351.

GOLDMAN, S., AUER, G., ERHARDT, K. & SELIGSON, U. (1987). Prognostic significance of clinical stage, histologic grade, and nuclear DNA content in squamous cell carcinoma of the anus. *Dis. Colon Rectum*, **30**, 444.

GOLIGHER, J. (1984). Carcinoma of the anal canal and anus. In *Surgery of the Anus, Rectum and Colon*, Chapter 20, 5th edition, p. 780. Ballière Tindall: London.

HEDLEY, D.W., FRIEDLANDER, M.L., TAYLOR, I.W., RUGG, C.A. & MUSGROVE, E.A. (1983). Method for analysis of cellular DNA content of paraffin embedded pathological material using flow cytometry. *J. Histochem. Cytochem.*, **31**, 1333.

HIDDEMANN, W., SCHUMANN, J., ANDREEFF, M. and 6 others (1984). Convention on nomenclature for DNA cytometry. *Cancer Genet. Cytogenet.*, **13**, 181.

KAPLAN, E.L. & MEIER, P. (1958). Nonparametric estimation from incomplete observations. *J. Am. Stat. Assoc.*, **53**, 457.

KOKAL, W., SHEIBANI, K., TERZ, J. & HARADA, J.R. (1986). Tumour DNA content in the prognosis of colorectal carcinoma. *JAMA*, **255**, 3123.

MANTEL, N. & HAENSZEL, W. (1959). Statistical aspects of the analysis of the data from retrospective studies of disease. *J. Natl Cancer Inst.*, **22**, 719.

NIGRO, N.D. (1987). Multidisciplinary management of cancer of the anus. *World J. Surg.*, **11**, 446.

QUIRKE, P., DIXON, M.F., CLAYDEN, A.D. and 4 others (1987). Prognostic significance of DNA aneuploidy and cell proliferation in rectal adenocarcinomas. *J. Pathol.*, **151**, 285.

SAS PROGRAM INSTITUTE (1986). *Supplemental Library Users Guide*, Version 5 edition. Cary, NC: SAS Institute Inc.

SCHUTTE, B., REYNDERS, M.M.J., BOSMAN, F.T. & BLIJHAM, G.H. (1985). Flow cytometric determination of DNA ploidy level in nuclei isolated from paraffin embedded tissue. *Cytometry*, **6**, 26.

SCOTT, N.A., RAINWATER, L.M., WIEAND, H.S. and 4 others (1987*a*). The relative prognostic value of flow cytometric DNA analysis and conventional clinicopathologic criteria in patients with operable rectal carcinoma. *Dis. Colon Rectum*, **30**, 513.

SCOTT, N.A., WIEAND, H.S., MOERTEL, C.G., CHA, S.S., BEART, R.W. & LIEBER, M.M. (1987*b*). Colorectal cancer: Dukes stage, tumour site, preoperative plasma CEA level, and patient prognosis related to tumour DNA ploidy pattern. *Arch. Surg.*, **122**, 1375.

TRIBUKAIT, B., GUSTAFSON, H. & ESPOSTI, P.L. (1982). The significance of ploidy and proliferation in the clinical and biological evaluation of bladder tumours: a study of 100 untreated cases. *Br. J. Urol.*, **54**, 130.

VINDELOV, L.L., CHRISTENSEN, I.J. & NISSEN, N.I. (1983). A detergent-trypsin method for the preparation of nuclei for flow cytometric DNA analysis. *Cytometry*, **3**, 323.

WOLLEY, R.C., SCHREIBER, K., KOSS, L.G., KARAS, M. & SHERMAN, A. (1982). DNA distribution in human colon carcinomas and its relationship to clinical behaviour. *J. Natl Cancer Inst.*, **69**, 15.